# The Benefits and Limits of Technological Advances in Glucose Management Around Physical Activity in Patients Type 1 Diabetes

**DOI:** 10.3389/fendo.2018.00818

**Published:** 2019-01-18

**Authors:** Sémah Tagougui, Nadine Taleb, Rémi Rabasa-Lhoret

**Affiliations:** ^1^Institut de Recherches Cliniques de Montréal, Montreal, QC, Canada; ^2^Département de Nutrition, Faculté de Médicine, Université de Montréal, Montreal, QC, Canada; ^3^Département des Sciences Biomédicales, Faculté de Médicine, Université de Montréal, Montreal, QC, Canada; ^4^Division of Endocrinology, McGill University, Montreal, QC, Canada; ^5^Endocrinology Division, Montreal Diabetes Research Center, Montreal, QC, Canada

**Keywords:** type 1 diabetes, exercise, continuous subcutaneous insulin infusion, continuous subcutaneous glucose monitoring, artificial pancreas, single-hormone, dual-hormone, closed-loop

## Abstract

Physical activity is highly recommended for patients living with type 1 diabetes (T1D) due to its varied health benefits. Nevertheless, glucose management, during and in the hours following exercise, represents a great challenge for these patients who most often end up leading a sedentary life style. Important technological advances in insulin delivery devices and glucose monitoring are now available and continue to progress. These technologies could be used to alleviate glucose management related to physical activity in T1D. Continuous glucose monitoring (CGM) helps patients observe the trends of glycemic fluctuations when exercising and in the following night to deal pre-emptively with hypoglycemic risks and treat hypoglycemic episodes in a timely manner. Insulin pumps offer the flexibility of adjusting insulin basal rates and boluses according to patient's specific needs around exercise. The artificial pancreas links CGM to pump through an intelligent hormone dosing algorithm to close the loop of glucose control and has thus the potential to ease the burden of exercise in T1D. This review will examine and discuss the literature related to physical activity practice using each of these technologies. The aim is to discuss their benefits as well as their limitations and finally the additional research needed in the future to optimize their use in T1D.

## Introduction

Regular physical activity (PA) offers many potential health benefits for individuals with type 1 diabetes (T1D) including improvements in insulin sensitivity and requirements, reduced risk of cardiovascular diseases and increased overall life expectancy (1). Despite these benefits, physical activity is practiced at a much lower frequency than recommended by patients with T1D (2) who may adopt unhealthy lifestyles worsening their cardiometabolic risk profile (3). The principal barrier for PA is the fear of hypoglycemia that is mainly driven by the inability to identify and/or implement effective strategies for hypoglycemia avoidance (2).

**Figure 1:**
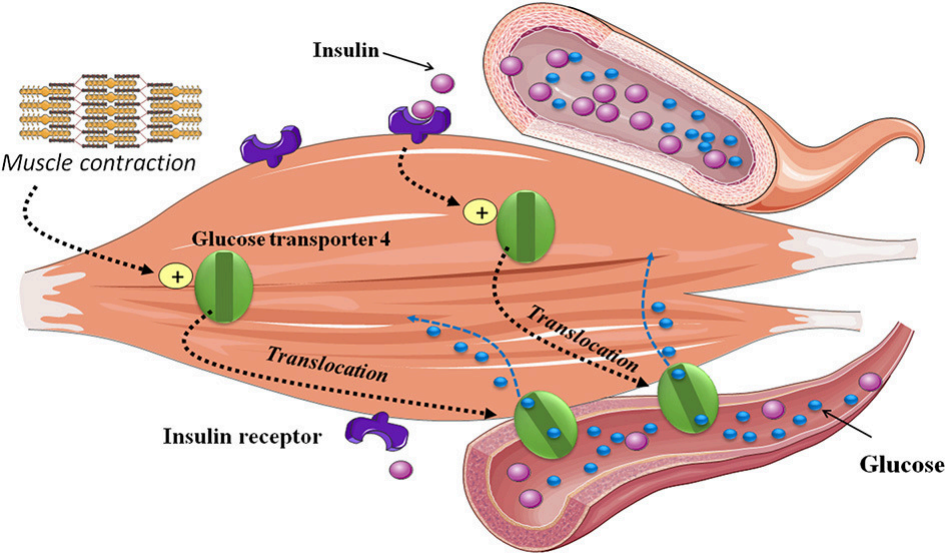
Skeletal muscle glucose uptake during exercise. Exercise increases insulin-stimulated glucose uptake in skeletal muscle. This process is regulated by the translocation of glucose transporter-4 glucose to the plasma membrane and transverse tubules. Both exercise and insulin utilize different signaling pathways, both of which lead to the activation of glucose transport.

**Figure 2:**
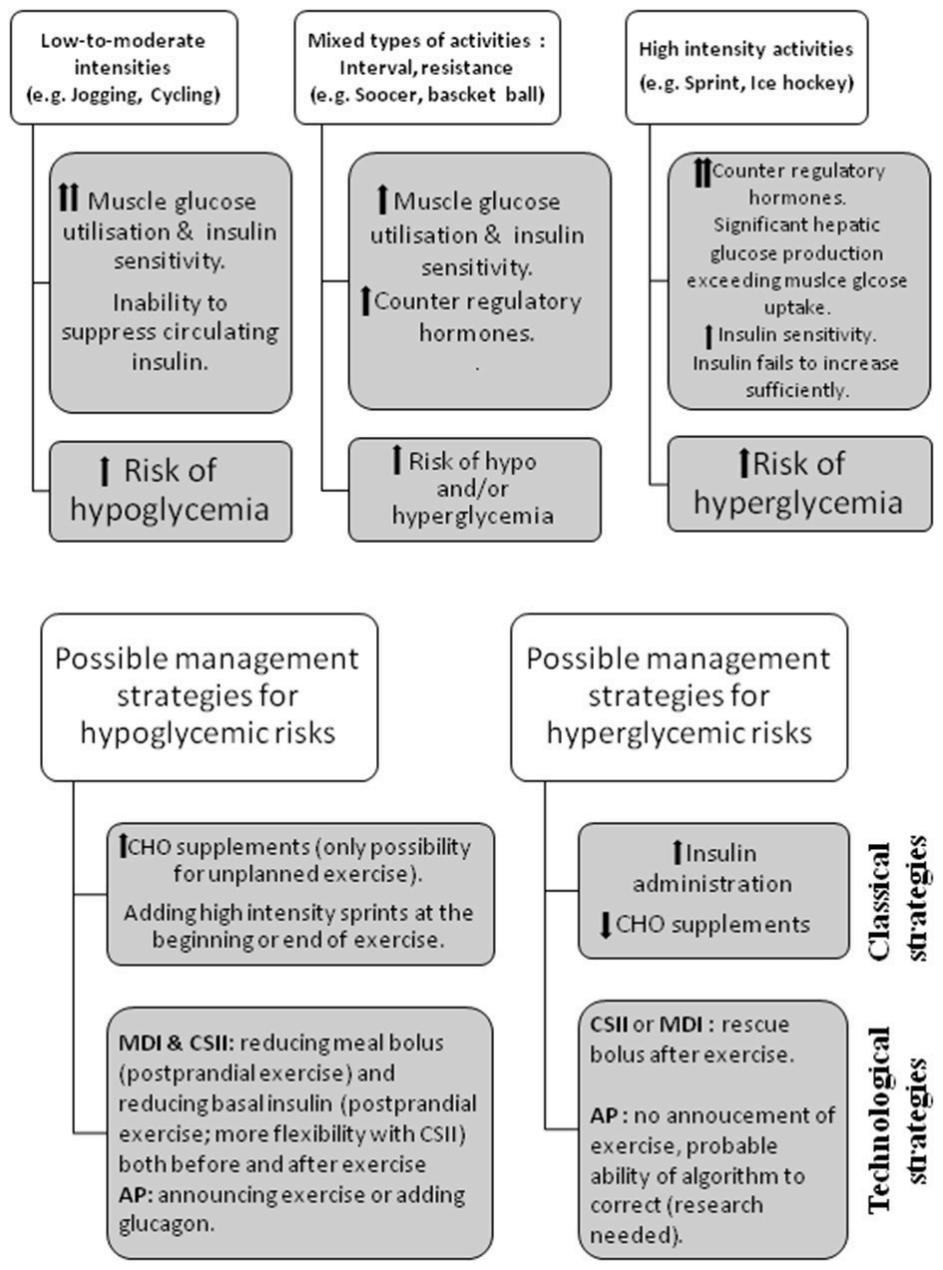
Physiological mechanisms related to hypo and hyperglycemia & strategies to limit exercise-induced hypo and hyperglycaemia in patients with type 1 diabete. CHO, carbohydrate; CSII, continuous subcutaneous insulin infusion; MDI, Multiple dose injection; AP, artificial pancreas.

As compared to people without T1D, the inability to reduce circulating insulin during and after exercise restricts hepatic glucose production concurrently to an enhanced glucose disposal rate into skeletal muscle. Skeletal muscle plays a considerable role in maintaining homeostasis of blood glucose. It uses glucose as a source of energy during dynamic exercise, and represents the major site for insulin-stimulated glucose uptake. Glucose is transported from blood into muscle fibers by the glucose transporter-4. This process is regulated by the translocation of glucose transporters-4 to the plasma membrane and transverse tubules under insulin and exercise-stimulated conditions (4) (Figure 1). Because of the disparity between glucose production and utilization (e.g., combined exercise and relative hyperinsulinemic conditions), hypoglycemia can occur during and in the hours following exercise (5). The type, intensity, duration, and distance to meals of exercise as well as the aerobic fitness are all important factors influencing glucose homeostasis (6, 7) (Figure 2). Therefore, aerobic, sprint, and resistance training can be responsible for wide variations in blood glucose responses (8, 9). While low to moderate aerobic exercise usually induces progressive glucose lowering, high intensity activities can trigger significant release of counter regulatory hormones (e.g., epinephrine and glucagon) causing rapid elevations in blood glucose levels (Figure 2). Exercise duration also affects glucose control for example, extended periods of exercise results in a higher rate of glucose disposal and thus increased risk of hypoglycemia. Hormonal counter-regulatory response (e.g., catecholamines, glucagon, etc.) to different types of prolonged exercise could be highly variable inter- and intra- individuals as shown in well-controlled T1D (10, 11). For very intense exercise it can trigger a short but large hepatic glucose output exceeding glucose utilization ability by other tissues resulting in transient but significant hyperglycemia (5) (Figure 2). Even with low intensity and realtively short duration exercise practiced during the day, an increase in insulin sensitivity can last up to 11–16 h post-exercise which combined with glycogen store replacement can increase the risk of late-onset or nocturnal hypoglycemia (Figure 2).

Guidelines for minimizing exercise-related hypo- and hyperglycemic risks exist but remain general (12). In practice, 3 main adjustments may be considered (13, 14): **1/** carbohydrate (CHO) supplementation (15, 16), particularly for unanticipated exercise and for prolonged exercise; **2/** premeal insulin-dose reduction (17, 18) provided exercise can be anticipated and undertaken within meal bolus insulin action and for patients using continuous subcutaneous insulin infusion (CSII: pump) for both post-prandial and post-absorptive periods using temporary basal insulin reduction; **3/** adding high intensity sprints in intervals, or resistance training at beginning or end of exercise sessions (9). However, even when one or combinations of these strategies are used, the right combination for a specific exercise is complex to establish and most patients still have wide exercise-related glucose fluctuations.

Remarkable progress has been achieved in technologies to improve and facilitate diabetes management including insulin pumps, continuous glucose monitoring (CGM), and external artificial pancreas systems. This review aims to discuss how technological advances could be used to alleviate the burden of glucose management during exercise in patients with T1D. Each of these technologies is reviewed to examine its benefits as well as limitations around exercise.

## Continuous Subcutaneous Insulin Infusion (CSII: Insulin Pumps)

In this section we aim to present an update of practical management strategies during exercise in patients using insulin pump therapy. The introduction in the 1980's of CSII therapy in the form of pump systems was an important landmark in technological advances in insulin delivery (19). Insulin pumps provides a flexibility in an attempt to mimic physiologic insulin delivery, infusing rapid acting insulin subcutaneously at preselected basal rates to cover a 24 h period in addition to insulin boluses at mealtimes or to correct hyperglycemia that are activated on demand by the patient (20).

New insulin pumps are small in size and endowed with many programming options that facilitate their use. Insulin pumps exist in various designs the main difference being patch or tubeless pump vs. using an external catheter. In patch pumps, a container housing the insulin ampoule attaches directly to the skin with a catheter directly under the patch and a controller communicates wirelessly with the patch pump. In contrast, with the conventional design, the pump holds the insulin reservoir and connects to the body through a tubule up to a subcutaneous insulin catheter (21). While for aquatic physical activity patch pump since it is waterproof and does not require disconnection, the ability to transiently disconnect the pump with offered by conventional pumps can be useful for some other activities with contact (judo, hockey, etc.).

CSII therapy is proven to improve glucose control in many patients with diabetes resulting in lower HbA_1c_ levels and frequency of hypoglycemia, it is however associated with higher treatment cost and require to be continuously worn (22–24). Furthermore, a change in basal insulin delivery rate around the time of exercise is only possible with insulin pumps; an approach that cannot be used in multiple dose insulin injections (MDI) regimens where basal insulin is injected once or twice daily at preset timings. Indeed, for physically active patients with T1D, CSII can be a preferred option to facilitate glucose regulation.

Mild to moderate intensity aerobic exercise is probably the most chosen type of physical activity by patients and entails a high risk of hypoglycemia as discussed above. Therefore, this type of exercise was included in most of the conducted studies that have looked up ways to reduce hypoglycemia in patients with T1D (5). When exercise is planned within 90–120 min post meal, a pre-meal insulin bolus reduction that is proportional to exercise duration and intensity has been backed-up by a couple of studies and thus endorsed consensus statements (5, 17, 18, 25). When an exercise session is practiced in close proximity to a meal without anticipation, which is very frequent in adolescents and children, or when a longer than expected activity occurs then the consumption of carbohydrates (CHO) is usually required (15, 16). The CHO rescue could be an effective strategy to improve performance for certain patients who wish to improve their performance but could be counter-productive for those who wish to lose weight.

A third approach that can be applied in insulin pumps users consists of reducing temporarily basal insulin rate. Franc et al. (26) have tested the efficacy of reducing hypoglycemia by decreasing or stopping basal insulin rate at exercise onset. Adult patients with T1D performed 30 min of either moderate or high intensity aerobic in a post-absorptive state (>3 h post-meal). No hypoglycemia events (glucose < 3.3 mmol/l) were recorded: when basal insulin was reduced: **1/** by 50% or 80% during moderate exercise (50% VO_2peak_) or **2**/ by 80% or pump stopped for the intense exercise sessions (75%VO_2peak_). Another relevant observation from this study is that late post-exercise hypoglycemia risk was similar to rest interventions with the implementation of an 80% basal insulin reduction for moderate exercise and with pump suspension for the intense exercise (26). Another study conducted in an adult population with T1D looked at the effects of basal insulin suspension at exercise onset on blood glucose levels during 40-min continuous (40–50% VO_2peak_) vs. circuit-based exercise. The authors reported that basal insulin suspension at the onset of exercise leads to a greater drop in glycemia during Continuous vs. Circuit-Based Exercise (27). Currently, studies comparing the effects of basal rate reduction during continuous exercise vs. interval exercise remain uncommon. Considering that these type of exercise have a different impact the blood glucose level in T1D patients, it will be important to test strategies to determine the optimal approach to reduce hypoglycemia during these 2 types of exercise.

The studies cited above (26, 27) showed that as compared to no action, significant reduction in insulin infusion at exercise onset is generally helpful in improving time spent with blood glucose levels in target ranges during exercise, but the risk of hypoglycemia remains largely present. Thus, other studies have thereafter focused on completely suspending insulin infusion. In a pediatric study (*n* = 10), hypoglycemia episodes and the drop in glycemia during a 40–45-min exercise sessions were similar between a pump-on vs. pump-off strategies (28). In 49 children and adolescents (8–17 years old) with T1D, the DirecNet Study Group were able to bring down the risk of hypoglycemia from 43 to 16% by a pump-off design at exercise onset; 60-min aerobic exercise performed 4 h after lunch (29). The drawback in this study was an increased risk of post-exercise hyperglycemia.

Although reducing insulin basal rate at exercise onset seems could help in attenuating the hypoglycemia risk, earlier timings might be needed for a better effect. This is mostly supported by the pharmacokinetics of available rapid acting insulin analogs; suggesting that decreasing insulin rates up to 90 min prior to exercise onset might be needed to sufficiently reduce circulating insulin levels during a post-absorptive activity (26, 30). Practically speaking, though, such early anticipation is not practical for a large fraction of patients. A recent study compared 3 more practical timings of 80% basal insulin reduction: 40 min, 20 min prior and at exercise onset (31). This study reiterated the fact that exercise-induced hypoglycemia is frequent (close to 50% of exercise sessions). Moreover, although some favorable trends were observed with the reduction at 40 min prior to exercise, hypoglycemia remained a frequent event with that timing. Similarly, McAuley et al. (32) reported that reducing insulin infusion by 50% 1 h prior to a 30-min exercise at moderate intensity did not reduce plasma insulin levels at exercise onset and did not fully prevent exercise-induced hypoglycemia. An earlier basal rate reduction is thus probably needed to have a more significant impact on hypoglycemic risk.

It should be noted that practicing physical activity does not increase the risk of hypoglycemia only during exercise but also in the following hours frequently including overnight a time at which hypoglycemia prevention, detection and treatment is harder. To mitigate post-exercise hypoglycemia, very few evidence-based data is available. We have reviewed the impact of bedtime snack on nocturnal hypoglycemic risk and highlighted the very low level of evidence of this widely recommended practice (33). Taplin et al. (34) showed that children on CSII could significantly reduce their nocturnal hypoglycemia risk by stopping insulin infusion during exercise and reducing its rate by 20% over the following night (between 21 h:00 and 03 h:00).

The available studies are generally of a small scale (summarized in Table 1) and mostly conducted in laboratory settings, but could still help shaping some guidelines for glucose management around exercise for patients using insulin pumps. For anticipated exercise, if exercise occur during meal bolus action a reduction of this bolus proportional to exercise intensity and duration is a reasonably well-validated strategy; for exercise undertaken in the post-absorptive period in patients using CSII, the best timing and amount of insulin reduction prior to exercise onset is still a pending question. In all situations (post-meal vs. post absorptive as well as CSII vs. MDI) different timings and percentages need to be tested in different types of exercise (e.g., aerobic vs. resistance vs. interval; duration; intensity; etc.). In the case of unanticipated exercise, although insulin suspension at exercise onset seems the best solution for the time being, related studies have tackled mainly continuous moderate intensity exercise sessions. An increased risk of hyperglycemia could be speculated for intense continuous or interval exercise with pump suspension and evidence-based data is lacking. In that situation, to correct post exercise hyperglycemia, a recent study validated the efficacy and safety of a correction bolus based on usual correction factor (35). Finally to prevent late post-exercise hypoglycemic risk a nighttime basal rate reduction could be useful strategy.

**Table 1:** Main continuous subcutaneous insulin infusion studies with reported exercise related conclusions.

**References**	**Participants, *n***	**Design, insulin adjustments**	**Exercise description**	**Study conclusion**
Franc et al. (26)	Adults, 20	Randomized, single-blind, single-center, crossover study:1/Moderate-intensity exercise:50% BRR at start of exercise (+2 h in recovery)vs. 80% BRR at start of exercise (+2 h during recovery)2/ Intense exercise:Pump suspended during exercisevs. 80% BRR	1/30 min aerobic cycling (50% of VO_2peak_) 2/30 min intense cycling (75% of VO_2peak_)	To limit the hypoglycaemic risk associated with 30 min of exercise 3 h after lunch, without CHO supplements, the best strategy seem to be to reduce BR by 80% or to stop the pump for moderate or intense exercise, or for moderate exercise 90 min after lunch, to reduce the prandial bolus (~50%) rather than the basal rate
McAuley et al. (32)	Adults, 14	Prospective, open-label, two-stage randomized crossover study:50% BRR 1 h prior exercise after single insulin BRR overnightvs. Rest condition(T1D maintained a constant BR)	30 min aerobic cycling (65–70% of maximum heart rate)	Halving the BR 1 h prior to exercise did not significantly reduce circulating free insulin when BG is in low-normal rangeWhen BG < 7.0 mmol/L before exercise, consider carbohydrate snack and BR >50%
Zaharieva et al. (27)	Adults, 12	Randomized and counterbalanced study:Suspended BR during aerobic exercisevs. Suspended BR during circuit exercise	40 min aerobic exercise of treadmill walking (~50% of VO_2max_) 40 min circuit exercise (mean intensity ~55% of VO_2max_)	With BR suspension, aerobic exercise is associated with a greater drop in BG compared withcircuit-based exercise
Roy-Fleming et al. (31)	Adults, 22	Randomized, 3-way crossover study:80% BBR 40 min before exercisevs. 80% BBR, 20 min before exercisevs. BRR at the start of exercise	45 min submaximal cycling (~60% of VO_2max_)	Decreasing BR by 80% up to 40 min before exercise onset is insufficient to reduce exercise-induced hypoglycaemia.
Admon et al. (28)	Children and adolescents, 10	Randomized, single-blind, crossover study:Pump on (50% BRR) during exercise.vs. Pump off (Suspended BR) during exercise.	40–45 min submaximal cycling (~60% of VO_2max_)	No difference in hypoglycemia episodes between pump on vs. pump off and no significant difference in the drop in glycemia.
DirecNet Trial group. (29)	Children and adolescents, 49	Randomized crossover study:Pump on (normal BR) during exercisevs. Pump off (suspended BR) during exercise.	Four 15-min intervals on the treadmill at a target heart rate of 140 bpm (interspersed with three 5-min rest breaks over 75 min), followed by a 45-min observation period	Discontinuing BR during exercise is an effective strategy for reducing hypoglycemia in children with T1D, but the risk of hyperglycemia is increased.
Taplin et al. (34)	Children and adolescents, 16	Randomized crossover study:Suspended BR during exercise flowed by 50% BRR for 45 min during the 3 conditions:20% basal rate reduction for 6 hvs. 2.5 mg oral terbutalinevs. No treatment	Four 15-min intervals on the treadmill a target heart rate of 140 bpm (interspersed with three 5-min rest)	The authors speculate that suspending BR during exercise and reducing BR for 45 min post-exercise, reduce the frequency of afternoon hypoglycemia was well as delayed hypoglycemia during the night. BRR was safe and effective in raising post-exercise nocturnal BG nadir and in reducing hypoglycemia

*BR, basal rate; BRR, basal rate reduction; BG, blood glucose*.

Further studies are clearly warranted to guide insulin dose adjustments with CSII use. Because of the large inter- and intra-individual variability in glycemic responses to exercise, recommendations can only serve as general starting points that will need to be individualized.

## Continuous Glucose Monitoring

With the difficulty of glucose management during and post exercise in patients with T1D due to rapidly changing levels and hypoglycemia risks, individuals must increase the frequency of glucose monitoring during exercise and the following recovery period. This can be very cumbersome and undesired by many patients, especially when based on capillary glucose measurements. However, the introduction of continuous glucose monitoring in the early 2,000 has had a great impact on facilitating glucose profiling and helping with diabetes management. With CGM, interstitial glucose is measured repeatedly (e.g., each 10 min) via a subcutaneous sensor linked to a skin applied transmitter that relays these readings wirelessly either to a matched insulin pump or to a separate receiver which can be a cell-phone. CGM provides detailed glucose profiling in contrast to the readings that are possible with capillary measurements and has proved its efficacy in improving diabetes management and reducing hypoglycemia rates (36–38).

For physical activity, CGM has helped in gaining better understanding of changing glucose levels during and particularly in the hours following different types and conditions of exercise [an aspect reviewed recently by Houlder et al. (39).

One of the first reports to demonstrate the utility of CGM during exercise was an observational study conducted in 25 adolescents (8–17 years old) during a 2-week sports camp (40). An algorithm of CHO consumption (8–20 g) was followed according to CGM alerts, tendencies and rates of glucose change. Out of 22 uses of the CHO intake algorithm after CGM trend arrows indicated rapidly dropping glucose levels, only 2 hypoglycemia (3–3.9 mmol/l) events occurred (9%). The rate of hypoglycemia was higher (38%) though when glucose was lower than 5 mmol/l at the time of the alert despite consuming 16 g CHO (40). A recent study has also shed the light on the efficacy of combining CGM with a decision support system (DSS) in managing diabetes (41). The CGM+DSS helped patients adjust their insulin and CHO intake through insulin bolus, exercise and automated insulin titration advice. In a cross-over design with a wash-out period, 24 adults (on CSII or insulin injections) spent 48 h at a research clinic using CGM+DSS vs. usual care (41). The protocol included 2 sessions of 45 min (three 15 min exercise with 5 min rest in between) of mild to moderate aerobic exercise. Less time was spent in hypoglycemia (< 3.9 mmol/l) with CGM+DSS during exercise (1.8 ± 2 vs. 3.8 ± 4.6%, *p* = 0.018). This improvement with CGM+DSS was observed despite significantly lower consumption of CHO before initiating and during exercise and reduced glucose variability in comparison to usual care (41).

Interestingly, patients with T1D and health professionals who attended a boot camp that included real time CGM, in-class teaching and supervised exercise sessions have identified real time CGM as the best learning tool about glucose changes during exercise (42). Patients reported that CGM helped improve glucose control by keeping it in target ranges during sports without needing extra capillary measurements (e.g., less fear of hypoglycemia as well as decreased intake of unnecessary CHO with CGM use) (42).

One possible limitation to CGM usage is a lower accuracy during exercise. This has been recognized in the literature and needs to be understood by patients and healthcare professionals. Among factors involved to explain this lower accuracy rapid blood glucose changes that accompany physical activity is probably a dominant factor. Such situations increase the lag time between blood and interstitial glucose values due to delay to reach equilibration between compartments (5). This CGM delay has been estimated to reach up to 15 min during exercise and could result in either over- or most frequently underestimation of blood glucose. For example, mean difference between CGM (Medtronic Guardian Real-Time system) and plasma glucose was 1.4 ± 0.8 mmol/L during continuous aerobic exercise implicating an overestimation of plasma glucose (43). On the other hand, an underestimation of blood glucose by CGM has been reported with resistance type of exercise (44).

Most manufacturers and studies report CGM accuracy with median absolute relative difference (MARD) of CGM relative to blood glucose (capillary or venous); reflecting an average deviation from the reference in either direction. MARD of two CGM devices Dexcom G4 Platinum and Enlite in reference to plasma glucose was evaluated at rest vs. exercise (continuous and interval) (45). MARD was increased from 13.3 (6.1–12.2)% at rest to 15.1 (9.4–26.8 %; *p* = 0,02) during exercise for Dexcom and from 11.9 (7.8–14.2)% to 14.1 (8.5–22.7%, *p* = 0.02) for Enlite (45). In this cross-over trial design, no significant differences in MARD were observed between continuous vs. interval exercise that were notably matched in total energy expenditure per patient (45), this is in congruence with another study comparing continuous moderate intensity and high intensity interval exercise sessions (46). On the other hand, significant differences in accuracy by MARD during continuous vs. interval exercise was reported by another group despite comparing the two types of exercise at similar intensities (47). Contrasting conclusions from these reports about CGM accuracy per distinct exercise types could be related to the respective studies design and small sample size. For example, including a pre-exercise snack (slower decline in blood glucose) or hypoglycemia correction with CHO are all factors that could affect the interpretation and comparison of MARD across different studies.

The overall accuracy of CGM devices during exercise is lower but still remains acceptable under exercising conditions. Patients are encouraged to follow the arrow trends in their GCM devices and set their hypoglycemia alarms to higher values to anticipate events (5). Future research efforts should thus consider more comprehensive analysis of CGM biases over the course of different types of exercise and not only reporting average over the whole exercise session. Studies should also report clear analysis of CGM accuracy during hypoglycemic episodes (onset to reduce occurrence and following correction to reduce possible overcorrection) preferably in comparison to capillary or venous reference values. The results of such analyses could then be translated into clinical messages to help patients choose thresholds when setting their alarms to optimize the use of this option while concurrently following CGM arrow trends.

In summary, CGM technology eases the challenge of glucose management during and after physical activity in patients with T1D but patients need to be educated about the lower accuracy of these devices during exercise.

## Artificial Pancreas Systems:

Further technological progress has been achieved by linking CGM to insulin pumps (48, 49) including sensor-augmented pumps (SAP), low suspend and predictive-suspend pump systems. In order to reduce hypoglycemia frequency, importance and length these technologies helps patients adjust their insulin treatment based on real-time feedback from the CGM function (50). These systems are technological steps along the way to closing the loop of glucose control with the artificial pancreas systems targeting both hypo and hyperglycemia.

The artificial pancreas (AP), equally referred to as closed-loop system, constitute to date the most advanced, and promising technology for insulin delivery in T1D (51). Readings from CGM are communicated every few minutes to hormonal dosing algorithm which dynamically commands changes in hormonal basal rates or boluses administered by subcutaneous infusion pumps. Clinical studies investigating artificial pancreas systems are increasing exponentially. These clinical trials mainly cover research on two AP versions; single-hormone AP (SH-AP) which delivers only insulin and dual-hormone AP (DH-AP) which delivers besides insulin mini-boluses of glucagon. Two approaches govern the addition of glucagon to AP, either to allow more aggressive insulin delivery while avoiding hypoglycemia and generally aiming for lower glucoses targets or a more conservative approach which uses glucagon only after suspending insulin for low blood glucose in attempt to prevent pre-emptive hypoglycemia (51). Around two-thirds to one-third is the proportion of SH-AP to DH-AP in the published literature with a lot of heterogeneity in terms of design, reported parameters, types of algorithms used and patient populations tested.

Two recent meta-analyses have clearly shown the clinical efficacy of AP systems in comparison to conventional or sensor-augmented insulin pumps (52, 53). Time spent with glucose levels in-target-range (most used definition is 3.9–10 mmol/l) was increased in both analysis by means of 10–15% which are equivalent to 2.5 and 3 additional hours in target per 24 h (52, 53). The percentage of time spent in hypoglycemia (< 3.09 mmol/l) was decreased by means of 1.5–2.5 %, equivalent to a decrease by 20 and 35 min over 24 h (52, 53). *Post-hoc* analyses showed an added benefit of DH-AP with an increase by +8.4 and +8.6% in time-in-target and a further decrease by 1.9 and 1.6% over 24 h in hypoglycemia (52, 53). Some of these studies included exercise in their protocol but their designs and outcomes were not primarily centered around exercise and/or did not specifically present data during exercise limiting the conclusions that can be drawn in relation to physical activity practice. For this reason, we will limit the discussions in the following sections to the clinical trials that specifically reported exercise related outcomes.

Clinical research in AP examining exercise span a spectrum from SH-AP studies adopting fully closed loop systems to those testing hybrid systems with required input from patients/research team to guide the algorithm. Ideally, fully closed loop AP would be the easiest especially in the case of unplanned exercise. The algorithm would then be expected to adjust insulin delivery solely based on changing glucose readings. On the other end of the spectrum, hybrid systems involve exercise announcement by the patient to the algorithm to adjust glucose target ranges (higher targets) and adopt a more cautious insulin delivery. In between the two ends, trials include addition of glucagon in DH-AP, use of exercise detectors (such as heart rate or movement) to guide the algorithm to self-adjust or combinations of these approaches.

The aim behind investigating these different strategies is to account for exercise-induced: 1-increases in insulin sensitivity and absorption from subcutaneous depot due to skin heat and movement, 2- delays in CGM due to rapid changes in blood glucose levels (5, 6). As discussed in the previous sections, the effect on glucose of different types, duration, intensity and timing of exercise need to be taken into consideration when examining AP studies around physical activity (6).

Good overall results were observed in a study conducted with unannounced 40 min exercise performed in a postprandial state as moderate and interval sessions in children and adolescents with T1D (54). Median time of glucose-in-target (3.9–10 mmol/l) was improved during exercise with SH-AP vs. standard insulin pump therapy; however, there were no differences in percentage of time in hypoglycemia ranges or in events requiring CHO replacement (54). In another study, unannounced exercise to SH-AP algorithm was examined during prolonged skiing activity (two sessions per day, 5 day camp) in a group of adolescents and compared to another matched group under sensor augmented pump (SAP) control (55). Although an overall benefit was seen with SH-AP vs. SAP per 24 h and overnight for time spent with glucose-in-target, this was not maintained during the pooled skiing sessions and the hypoglycemia events requiring CHO correction (Table 2) (55).

**Table 2:** Main artificial pancreas studies with reported exercise related outcomes.

**References**	**Participants, *n***	**Design, comparators**	**Exercise description**	**Main outcomes**
Dovc et al. (54)	Chidren and adolescents, 20	Cross-over randomized SH-AP vs. CSIICSII: pump stopped during exercise and basal rate reduced by 20% for 4 h post exercise AP: exercise unannounced	Two exercise types of 40 min, postabsorptive Cycle ergometer Moderate 55% VO_2_ max Interval 55%/85% VO_2_ max	No difference in time spent in hypoglycemia range during exercise Better median time-in-target (3.9–10 mmol/l) with AP for interval: 75.3 (IQR: 66.6–92.9) % vs. 68.4 (52.1–77.2) %, *p* = 0.02 and for moderate 80.9 (64.3–92.2)% vs. 68.1(59.1–83.6)%, *p* = 0.09 No difference in hypoglycemia events requiring CHO correction over the whole study period 22 h (7 AP vs. 8 CSII)
Breton et al. (55)	Adolescents, 32 (16 per group)	Controlled randomized SH-AP vs. SAP AP: exercise unannounced	5 days skiing camp Two skiing sessions/day 9 h:30 to 12h:00 13 h:00 to 16h:00	No difference in mean time-in-target (3.9–10 mmol/l) between AP and SAP during skiing sessions: 63.2 ± 31.1% vs. 62.8 ± 31.4%, *p* = 0.60 less time spent below 3.9 mmol/l in AP: 1.4 ± 1.6% vs. 2.3 ± 6.4%, *p* = 0.04 No difference in hypoglycemia events or CHO requiring incidences
Patel et al. (56)	Adolescents and adults, 12	Cross-over randomized SH-AP vs. SH-AP + snack Snack: 15–30 g CHO before and midway during exercise for PG < 8.3 and > 8.3 mmol/l respectively AP: exercise unannounced	Four 15 min 60–75% of maximal heart rate, with three 5 min in between rest and 30 min recovery Treadmill ergometer	15 g CHO were given to 75% of participants prior to exercise and to all of them midway through exercise Hypoglycemia events requiring CHO were 3 in AP vs. 0 in AP+snack Mean PG at end of session was 10 ± 0.5 vs. 6.1 ± 0.9 mmol/l in AP+snack vs. AP, *p* = 0.004
Jacobs et al. (57)	Adults, 21	Cross-over randomized DH-AP with adjustments vs. DH-AP without adjustments vs. SAP(with allowed adjustment per patient) Adjustment description at start of exercise: Insulin suspended for 30 min then reduced by 50% for 1 hr Glucagon doubled for 90 min AP: exercise announced at start	45 min exercise at 60% of maximal heart rate 2 h post breakfast Treadmill ergometer	Mean time in hypoglycemia (< 3.9 mmol/L) was 0.3 (95%CI: −0.1%,0.7)% with adjustment, 3.1 (0.8–5.3)% with no adjustment and 0.8 (0.1–1.4)% with SAP, adjustment vs. no adjustment *p* = 0.001, adjustment vs. AP *p* = 0.16 No difference for time spent in target among the three trial arms
Taleb et al. (58)	Adults, 17	Cross-over, randomized, 4 arms SH-AP vs. DH-AP during continuous and interval exercise AP: exercise announced 20 min prior to start	60 min exercise, postabsorptive continuous session: 60% VO_2_ max Interval session: 2 min interval alternating 50%/85% Cycle ergometer	Benefit of DH-AP vs. SH-AP seen with both types of exercise, more glucagon used during continuous than interval For pooled exercise sessions, DH-AP vs. SH-AP: median time PG in target was 100 (IQR: 100–100)% vs. 71.4 (53.2–100)%, *p* = 0.003 and in hypoglycemia (< 3.9 mmol/l) was 0% vs. 11 (0–46.7)%, *p* = 0.0001 3 vs. 15 hypoglycemia events corrected with CHO in DH-ap vs. SH-AP
Breton at al. (59)	Adults, 12	Cross-over, randomized, 2 arms SH-AP+heart rate vs. SH-AP AP: exercise detected	mild intensity exercise reaching 9–10 on exhaustion borg scale Postprandial Cycle ergometer	less glucose decline in SH-AP with heart rate at −0.3 vs. −1.6 mmol/l, *p* = 0.02 less hypoglycemia events with heart monitoring but not significant (0 vs. 2)
Jacobs et al. (60)	Adolescents, 18	Cross-over, randomized, 2 arms SH-AP+heart rate vs. SH-AP AP: exercise detected	45 min exercise, postabsorptive Three 15 min episodes of exercise with HR reaching 140 beats/min separated with 5 min rest Cycle ergometer	Mean time spent in hypoglycemia (< 3.9 mmol/l) was 0.5 ± 2.1 % in SH-AP+HR. vs. SH-AP 7.4 ± 12% (*p* = 0.03) No difference in hypoglycemia events which were overall low in both arms
Castle et al. (61)	Adults, 20	Cross-over, randomized, 4 arms DH-AP, SH-AP, Predictive low glucose suspend and Current care (pre-exercise insulin adjustments were allowed)Both DH-AP and DH-AP had exercise detection algorithms with inputs from heart rate and accelerometer (ZephyrLife BioPatch) AP: exercise detected then confirmed to algorithm	45 min exercise 2 h post lunch at 60% of VO_2_ max performed on days 1 and 4 in research lab while the rest was free living at home	Time in hypoglycemia (< 3.9 mmol/l) during exercise was lowest with DH-AP 3.4 ± 4.5 % vs. 8.3 ± 12.6% with SH-AP (*P* = 0.009) vs.7.6% ± 8.0% with predictive low glucose suspend (*P* < 0.001) vs. 4.3 ± 6.8% with current care (*P* = 0.49), (*p*-values are DH-AP against the other arms) lower hypoglycemia events requiring CHO correction was seen with DH-AP over the 4 day period

*DH-AP, dual-hormone artificial pancreas; SH-AP, single-hormone artificial pancreas; CSII, continuous subcutaneous insulin infusion; SAP, sensor-augmented pump, CHO, carbohydrates*.

With unannounced exercise to SH-AP, adding a snack (15 or 30 g CHO for PG < 8.3 mmol/l and > 8.3 mmol/l, respectively) prior and midway through moderate intensity aerobic exercise could prevent drops in plasma glucose and hypoglycemia requiring correction (0 in AP with snack vs. 3 in AP without snack) (56). Authors in this study did not report percentages of time-in-target, hypo- or hyperglycemia, but around half of the participants ended up with plasma glucose between 10 and 13 mmol/l in SH-AP+snack. These results support the use of a simple snacking strategy to avoid exercise-induced lowering of PG while on AP (56). However, snack consumption may be undesired given the increased prevalence of the metabolic syndrome in patients with T1D who frequently practice exercise with in weight loss or maintenance objectives (3).

Other strategies to improve AP performance around physical activity consisted of examining the effect of glucagon addition through DH-AP systems and exercise announcement to the algorithm. Jacobs et al. tested if announcing physical activity to their DH-AP algorithm by adjusting its insulin and glucagon dosing at the start of a 45 min aerobic moderate intensity exercise could improve glucose management in the following hours (57). Insulin was suspended for 30 min and reduced by 50% for the following 60 min while glucagon was doubled for 90 min from start of exercise (57). Less time was spent in hypoglycemia with adjustment to DH-AP by 2.8% vs. no adjustment (*p* = 0.001) and no difference vs. sensor augmented pump (*p* = 0.16). The authors observed a similar time spent with glucose-in-target between the three arms (57).

Another head-to-head SH-AP to DH-AP comparison in which insulin dosing algorithm is similar in order to specifically investigate the additional benefit of glucagon incorporation in AP during exercise (58). Two types of exercise sessions consisting of 60 min of continuous and interval exercise were performed in the postprandial state under both SH-AP and DH-AP on 4 separate visits (58). Exercise was announced 20 min prior to its start which resulted in changing the target glucose level from 5.3 to 8.3 mmol/l till the end of the 60 min session. Overall, with DH-AP, median time spent with glucose-in-target was increased by 28.6% (*p* = 0.003) and time in hypoglycemia (PG < 3.9 mmol/l) was decreased by 11% (*p* = 0.0001) in comparison to SH-AP. The number of hypoglycemia events requiring CHO treatment were also reduced (3 in DH-AP vs. 15 in SH-AP), all showing an added benefit of glucagon in AP during exercise (58).

An alternative to directly announcing exercise sessions to an AP algorithm was sought by some groups using exercise detectors such as heart rate monitors or accelerometers. The idea behind exercise detection and indirect announcement is to relieve patients from active inputs especially during unplanned and unknown activity intensities. Such systems would be particularly interesting to investigate in youngsters whose activity level is often unpredictable making them at high risk for both hypo- and hyperglycemia. Breton et al. were among the first to study the feasibility of adding heart rate monitoring to a SH-AP in 12 adults performing mild 30 min exercise sessions (exhaustion at 9–10 on Borg scale) (59). Whenever the heart rate exceeded 125% of its resting value, the algorithm was informed manually which would result in less aggressive insulin delivery and modification of hypoglycemia risk (59). A significant decrease in glucose decline during exercise was noticed when adding HR monitoring (−0.3 vs. −1.6 mmol/l, *p* = 0.02), but only a mild non-significant effect was observed in terms of hypoglycemia events (59). Similar results were observed by Jacobs et al. with their SH-AP with a heart rate monitor connected via blue tooth which informed the algorithm of exercise of physical activity when heart rate exceeded 125% of resting levels (60). This triggered a change in glucose target from 6.2 to 7.8 mmol/l and subsequent insulin delivery by the algorithm. A decrease in time spent in hypoglycemia (< 3.9 mmol/l) was significantly reduced with heart rate signal integration to SH-AP 0.5 ± 2.1% vs. 7.4 ± 12.5% (*p* = 0.028) without an effect on the incidence of hypoglycemic events which was however low in both arms (60).

A combination of different strategies was also tested. Recently, an interesting study was performed in adults comparing DH-AP and SH-AP that adapt to exercise using wearable sensors with predictive low glucose suspend and current care during and after exercise (61). Both AP systems had an integrated algorithm for exercise detection that receives input from heart rate monitor and accelerometer (the ZephyrLife BioPatch). Once exercise was detected, the participant was asked by the algorithm to confirm it and the changes to insulin and glucagon were similar to what is described above for the study by Jacobs et al. (57). Additionally the DH-AP was adaptive with adjustments to glucagon delivery at earlier timings and higher glucose levels on subsequent days 2–4 in comparison to day 1. Day 1 and 4 were partly spent at research clinic and the rest at home, formal 45 min exercise sessions 2 h post lunch were performed at 60% VO_2max_ during clinic stay (day 1 and day 4) (61). The lowest time spent in hypoglycemia (< 3.9 mmol/l) during exercise till next meal was with DH-AP 3.4 ± 4.5 vs. 8.3 ± 12.6% with SH-AP (*p* = 0.009) vs. 7.6 ± 8.0% with predictive low glucose suspend pump (*p* < 0.001) vs. 4.3 ± 6.8% during usual care (*p* = 0.49) (61). Number of hypoglycemia events requiring CHO consumption was also lowest with DH-AP over the whole study period with a mean of 0.8 ± 0.7 treatments per day vs. 1.7 ± 1.4 with SH-AP (*p* = 0.004), 1.3 ± 1.3 with the predictive low glucose suspend pump (*p* = 0.065), and 1.5 ± 1.2 with usual care (*p* = 0.10) (61).

The AP studies that specifically tackled glucose control in relation to exercise are still heterogeneous, small in size and do not cover all exercise scenarios (Table 2 summarizes the discussed trials). Most to date cover moderate intensity exercise performed in the post-absorptive state (Table 2). Nevertheless, they highlight the positive impact of artificial pancreas systems around exercise. AP is still an emerging technology and many future trials at large scale and in outpatient settings are needed in general and around exercise in particular.

Directly announcing exercise seems to still be needed for optimized results but the timing of the announcement from the start of exercise maybe an area to explore in future studies particularly for postprandial exercise when meal insulin boluses are active. Exercise detection by sensors is an interesting avenue particularly for children and adolescents living with T1D but adds the burden of wearing additional devices necessitating active research efforts in the future to develop small sensors integrated to the artificial pancreas itself.

Glucagon clearly shows an added benefit but the complexity of adding an additional chamber and material needs to be weighed against the additional hypoglycemia benefit. Therefore, future research trails should be designed to carefully identify patients who are most in need of glucagon and show high rates of exercise-induced hypoglycemia. While SH-AP currently reach the market in various countries, DH-AP are not expected to be commercialized in the near future since stable glucagon formulations are not yet available for use but promising research is underway. Clinical trials with DH-AP may still be conducted with the commercially available glucagon used for severe hypoglycemia treatment but needs to be reconstituted every 24 h (62). Meanwhile, another pressing aspect is proving the safety profile of chronic glucagon use in its different formulations or analogs given its multisystemic effects in humans (63).

## Conclusions

Technological advances have endowed individuals with T1D with important tools to help them better manage their blood glucose during exercise mainly allowing more secure conditions with reduced hypoglycemia risks. Some limitations to the different technologies have been detailed in this review and future research areas that need to be explored have been highlighted as well. The hope is that optimizing the use of these different technologies during exercise will encourage the majority of patients with T1D to regularly engage in physical activity.

## Author Contributions

ST, NT, and RR-L conceived the study design and content. ST and NT drafted the manuscript which was critically reviewed by RR-L.

### Conflict of Interest Statement

RR-L has the following to declare: RR-L has the following to declare: Research grants from Astra-Zeneca, E Lilly, Merck, NIH, Novo-Nordisk, Sanofi-Aventis; Consulting/advisory pannel with Abbott, Amgen, Astra-Zeneca, Boehringer, Carlina Technology, Eli Lilly, Janssen, Medtronic, Merck, Neomed, Novo-Nordisk, Roche, Sanofi-Aventis; Honoraria for conferences received from Abbott, Astra-Zeneca, Boehringer, E Lilly, Janssen, Medtronic, Merck, Novo-Nordisk, Sanofi-Aventis; consumable gift (in Kind) from Abbott, Animas, Medtronic, Roche; unrestricted grants for clinical and educational activities from Eli Lilly, Lifescan, Medtronic, Merck, Novo Nordisk, Sanofi; patent for T2DM risk biomarkers, catheter life & artificial pancreas; Purchase fees related to artificial pancreas from Eli Lilly. The remaining authors declare that the research was conducted in the absence of any commercial or financial relationships that could be construed as a potential conflict of interest.
